# Elderly Dementia Needs Better Care in Post-COVID-19 Era

**DOI:** 10.14336/AD.2022.1122

**Published:** 2023-06-01

**Authors:** Lili Chen, Ayae Kinoshita, Hong Li

**Affiliations:** ^1^Shengli Clinical Medical College, Fujian Medical University, Fuzhou, China.; ^2^Fujian Provincial Hospital, Fuzhou, China.; ^3^Human Health Sciences, Kyoto University School of Medicine, Kyoto, Japan.

**To the Editor,**

A recent paper reported a machine learning study on the use of multiple drugs in patients with dementia. As many of these patients were already taking drugs to treat the underlying disease, the addition of more drugs after a diagnosis of dementia increased the potential complications, which made their management much difficult [1]. Nevertheless, the patients in this study were enrolled before the COVID-19 pandemic began [1, 2]. Therefore, this report may give certain references but seemed not sufficient for their drug management during or after the pandemic, as the problems associated with drug management became more complex and challenging after this study. Indeed, drug management and its associated complications deserve attention, but the situations changed as the COVID-19 pandemic lasting almost three years. As the pandemic nears its end, we call for better care for patients with dementia in the post-pandemic era.

First, patients with dementia have not been effectively controlled during the pandemic in terms of drug treatments and management of drug complications, and this is likely to bring great challenges with post-pandemic comprehensive management of these patients. Social resources are projected to be insufficient in the post-pandemic era, and the situation becomes exacerbated with the incoming socioeconomic recession [3]. For example, the right of dementia patients to receive COVID-19 treatment was disrupted in Japan, and patients in institutions were not easily transported to hospitals and were retained in institutions instead; in addition, their right to maintain cognitive functions by going out even if nursing homes are closed due to COVID-19 was violated as well. Thus, many patients with dementia have experienced worsening of their condition due to inadequate medical care[4]. Drug shortages may have become a growing global problem[5]. These shortages will result in the inability to effectively control the condition of dementia patients. Besides medicines, long-term and effective nursing care is also instrumental in disease control. The pandemic has led to ineffective nursing care and exacerbated the condition of patients with dementia. Thus, more and better medical services are required in the post-pandemic era [6]. Furthermore, the pandemic and different forms of dementia worsen each other [7]. The pandemic increases morbidity and mortality in patients with dementia [8]. Patients with dementia are more susceptible to COVID-19 infection, resulting in an increased risk of getting infected, increased severity of the infection, and increased mortality from the disease [9]. In addition, COVID-19 has profoundly affected the healthcare system, and we have to make corresponding changes for the management of patients with dementia in the post-pandemic era. For example, the nursing management model may shift from the traditional healthcare model to an innovative home care model to decrease caregiver burden and allow for sustained, long-term care [10]. Telemedicine services based on information and communication technology (ICT) would also help with dementia management [11]. Taken together, the above factors negatively affect dementia patients and their management. Thus, effective strategies and policies are urgently needed for their management in the post-pandemic era.

Furthermore, the pandemic has caused physical and psychological distress to dementia patients, making their management more complicated in the post-pandemic era. Besides increasing the morbidity and mortality of patients with dementia [8], the COVID-19 pandemic can also lead to the development or worsening of neuropsychiatric disorders because of lockdown measures and social isolation [12]. Even though some countries have made significant progress in containing the pandemic, the mental health crisis of these patients is likely to be continue into the post-pandemic era [13]. Their neuropsychiatric symptoms may significantly worse [14]. In detail, the risk of depression, social isolation, and even suicide continued to rise during the COVID-19 pandemic [15]. A study found that long-term loneliness and social isolation resulted in a 49%-60% risk of developing dementia, and this risk is markedly higher than of those who were not lonely or socially isolated; patients having dementia with lack of primary care during the pandemic suffered from limited support and social interaction [16]. Furthermore, the deterioration of cognitive function in patients with dementia caused by the pandemic was associated with increased psychological distress in caregivers [17]. Thus, more widespread and better medical care is required for these patients in the post-pandemic era as their physical and mental health has been deeply negatively affected by the pandemic [4]. Therefore, focusing on medication management alone is not sufficient. The psychological crisis of patients with dementia brought upon by the pandemic should not be ignored. We emphasize that a multidisciplinary, multi-timeline, and multidimensional approach involving a collaborative effort of mental health professionals, social workers, and volunteers should be considered to provide ongoing mental healthcare for patients with dementia. It is necessary to formulate specific norms for psychological rehabilitation, including specific and feasible rehabilitation suggestions and operational methods, and establish public psychological crisis response measures to promote public spirit and mental health in the post-pandemic era.

Taken together, the pandemic has complicated the management of dementia patients. As we reach the end of the pandemic, the impact of multiple adverse factors would inevitably highlight many gaps in the management of dementia patients. Therefore, we would like to emphasize the need for better care for dementia patients in the post-pandemic era, such as formulating dementia care guidelines, improving healthcare using smart pill boxes for medicine reminder and monitoring system, establishing a long-term effective homecare system based on ICT, and building a public dementia crisis management platform. These measures will help ensure good care of patients with dementia in the post-pandemic era.
